# The emergence of altruism as a social norm

**DOI:** 10.1038/s41598-017-07712-9

**Published:** 2017-08-29

**Authors:** María Pereda, Pablo Brañas-Garza, Ismael Rodríguez-Lara, Angel Sánchez

**Affiliations:** 1 Grupo Interdisciplinar de Sistemas Complejos, Departamento de Matemáticas, Universidad Carlos III de Madrid, 28911 Leganés Madrid, Spain; 2 Middlesex University London, Department of Economics, Business School, Hendon Campus, The Burroughs, London, NW4 4BT United Kingdom; 3 Institute UC3M-BS of Financial Big Data, Universidad Carlos III de Madrid, 28903 Getafe, Spain; 4 Institute for Biocomputation and Physics of Complex Systems (BIFI), University of Zaragoza, 50018 Zaragoza, Spain

## Abstract

Expectations, exerting influence through social norms, are a very strong candidate to explain how complex societies function. In the Dictator game (DG), people expect generous behavior from others even if they cannot enforce any sharing of the pie. Here we assume that people donate following their expectations, and that they update their expectations after playing a DG by reinforcement learning to construct a model that explains the main experimental results in the DG. Full agreement with the experimental results is reached when some degree of mismatch between expectations and donations is added into the model. These results are robust against the presence of envious agents, but affected if we introduce selfish agents that do not update their expectations. Our results point to social norms being on the basis of the generous behavior observed in the DG and also to the wide applicability of reinforcement learning to explain many strategic interactions.

## Introduction

In spite of the many fundamental issues that humanity still faces both at local and global scales, human society has proven capable of taking our species to levels of adaptation and success unrivaled in the animal world^1^. Research in evolutionary psychology and anthropology suggests that human beings are especially social mostly because they are especially cooperative^2, 3^. One of the main mechanisms behind such an ultra-cooperative behavior is expectations, that promote prosocial behavior through the willingness to fit in the group (to conform to the expectations the group has about oneself^4^) and/or to avoid punishment (for not following their expectations^5^). According to Bicchieri^6^, social norms are indeed governed by both empirical expectations (what we believe others will do) and normative expectations (what we believe others believe we will do). Thus, expectations which drive behavior become social norms^6^ to which most people conform, leading to an overall cooperative performance of the society^7, 8^. Social norms can serve to choose among different Nash equilibria in social complex environments –games– where individuals face strategic interactions^9, 10^ and drive behavior in non-strategic settings where individuals can choose actions depending on their expectations of others and the degree to which these actions are seen appropriate^11^.

A particularly well-suited framework to study expectations is the dictator game (DG for short), which has provided a large body of experimental evidence on altruistic behaviour in the lab during the last thirty years^12, 13^. The DG is a simple one-shot game with two players: the first one (the dictator) is invited to divide a specified amount between herself and the second player (the recipient). The dictator may divide the pie in the manner she sees fit, while the recipient is not permitted to make any claim to the money. Theoretically, self-centered preferences predict that the dictator keeps all the pie and the recipient receives nothing; hence, any positive donation can be interpreted as proof of generosity. Contrary to the self-centered prediction, Engel’s meta-analysis^12^ shows that a huge number of individuals do offer nonzero, often sizeable portions of the pie to the recipient. On average, subjects donate between 20–30% of the total pie with a non-trivial fraction of subjects choosing an equal split. Interestingly, some authors argue that this is indeed a lower bound for generosity given the absence of social context within a lab experiment^13–18^.

Expectations in the DG have been recently studied in a series of experiments that allowed to probe the influence of different social factors on the observations^19^. Specifically, we have found that even if we elicit expectations from people in different roles, or from external observers of the social interaction, or from subjects socially distant because they refer to a previous experimental session, or when the money at stake is large, we always find that people expect generous behavior. In fact, a majority of people expects a fair split and only about 10% of the subjects predict they will receive nothing. On the other hand, people have a behavior that is very correlated with their expectations, which supports the role of expectations in the formation of social norms (see also refs 11, 20 and 21).

In this paper, we model the formation of people’s expectations in terms of learning from own experience, and in particular we focus on expectations in the DG in order to validate our model by comparing to the large amount of available experimental evidence. We model behavior by assuming that people’s decisions are based on what they expect and on what they observe. Subjects are thus endowed with *aspirations* that reflect what they expect to gain from any interaction. In our setting, aspirations of subjects coincide in value with expectations about the donations they will receive as recipients; while when acting as dictators, we will posit that their donations are such that they keep the money that corresponds to their expectations [see Eq. (4 below)]. The fraction of the pie that recipients receive from dictators is compared with their aspiration level; when the donation is larger (than expected) then the stimulus is positive and leads to higher aspirations in the future and vice versa. This process is called *learning*. On the other hand, current decisions are “affected” by previous interaction with other players. Thus, any donation received by recipients has an influence in what they will donate in future; we call this effect *habituation* or *herding*. Most importantly in our setting, and in absence of noise, donations are bounded by aspirations, in the sense that subjects cannot exceed their own aspiration level when making a donation.

Our model is akin to other theoretical settings in which observed behavior and norms influence behavior. The work of Andrighetto *et al*.^8^, for example, develops an agent-based model in which contributions to a public good are affected by the norm salience, which is updated upon observing the contribution of other members and the past punishment decisions that may include normative messages or judgments on whether such contributions are viewed appropriately (see refs 22 and 23 for other models of social norms and refs 24 and 25 for experiments where subjects can express their disapproval). While subjects also learn form past interactions in our model, a key difference between our models is that we focus on a non-strategic interaction in which subjects update their expectations about generosity and this possibly affects their donations. Hence our paper complements the empirical evidence in ref. 11 where generosity seems to be affected by the social norms. Our contribution is to show that these norms can emerge as the result of updating expectations and aspirations that affect giving; i.e., we consider a dynamic model of reinforcement learning.

As we will show, the model summarized above leads to the following results: For any value —positive and smaller than one— of the learning and habituation parameters we find that an overhelming majority of the players donate about 30% of the pie and, consistently, they expect to receive 30% of the pie as well. There is almost no heterogeneity in the donation of dictators; in order to quantify this result, we have computed the Gini coefficient^26, 27^ to measure the degree of diversity in the donations, and found that it is close to 1. Therefore generosity emerges as social norm with almost no deviant subjects. It is quite remarkable that the observed average practically mimics the average result shown in the meta-analysis of 200 dictator game experiments^12^. However, its also important to have in mind that experiments with humans provide certain degree of variability of responses. To capture this heterogeneity, we also consider a stochastic version of the model where subjects with certain probability do not follow the social norm. While small noise does not impact substantially on results (average donation, 〈*d*〉 = 0.37, Gini coefficient of the distribution of donations, G = 0.85) we find that large noise generates a distribution that replicates Engel results in both average and heterogeneity (〈*d*〉 = 0.27; G = 0.71). The results about expectations are close to those found in our earlier experimental work^19^. We will also show that the results are robust against the existence of envious individuals, but they are very much affected by the presence of selfish individuals or free-riders, that never change their expectations and, consequently, their behavior.

Thus, our main message is that learning may explain the emergence and the adherence to a social norm in the society, and that this is indeed confirmed by the successful replication of the experimental results with human in many different environments. In fact, a necessary condition to recover the experimental results is just to let subjects make mistakes and also to be ready to learn. In the following sections we introduce our model in detail, present the results supporting this conclusions, and close the paper by discussing their implications.

## Methods

Let us consider *N* individuals interacting through a game. The game chosen is the Dictator Game (DG), a two-players degenerate game where the dictator player has to decide how to split an endowment Φ between herself and her partner. The recipient is passive and can only accept the donation. In the model, individuals play DG games iteratively but they change partner (and possibly role) every round: Each time step, pairs of individuals are randomly chosen among a population of *N* agents, and roles (dictator *D*/recipient *R*) are randomly assigned.

The update of strategies is performed each time step, after individuals have played one DG. In this game, we define strategy as the quantity a dictator is going to donate, i.e., the donation *D*_*i*_ of player *i* (in other words, strategies directly determine actions). Instead of using traditional strategy updating rules such as proportional imitation or Moran-like rules^28^, in our model individuals make decisions based on experiential induction, i.e. they update their strategies by reinforcement learning. To that end, we have developed a modification of the classical Bush-Mosteller (BM) algorithm^29^ (see also refs 30 and 31). In our model, only recipients, as a result of the game (dictator decisions), update their strategy to be used the next time they play the role of dictator. This intends to represent the fact that, after receiving a donation, agents update their expectations taking into account how much dictators gave in past games, and then use those expectations to decide on how much they themselves donate next time they act as dictators.

In detail, the algorithm works as follows: As in the original proposal, individuals have an aspiration level *A*_*i*_ (their expectations), representing the proportion of the endowment they expect to receive when playing as a recipient. Each individual *i* playing *R* (recipient) receives a stimuli ${s}_{i}^{R}\in [-1,1]$ as a consequence of her dictator’s decisions. When the difference between the donation received (payoff *π*_*i*_) and her aspiration level is positive, recipients receive a positive stimuli, and vice versa, according to

1 $${s}_{i,t}^{R}=\{\begin{array}{ll}({\pi }_{i,t}-{A}_{i,t})/({\rm{\Phi }}-{A}_{i,t}) & {\rm{if}}\,{\rm{\Phi }}\ne {A}_{i,t},\\ 0 & {\rm{if}}\,{\rm{\Phi }}={A}_{i,t},\end{array}$$

where Φ is the total amount to split among the players.

The stimuli, if positive, increases the willingness to earn more in the next encounter (meaning: “*I got more than I expected so I should expect to receive more*”), and vice versa, affecting future expectations. This effect is moderated by a learning rate *l* ∈ [0, 1] that balances the contribution of past experience. The expression for the change in the aspiration level is then

2 $${A}_{i,t+1}^{R}=\{\begin{array}{ll}{A}_{i,t}^{R}+({\rm{\Phi }}-{A}_{i,t}^{R})l{s}_{i,t} & {\rm{if}}\,{s}_{i,t}^{R}\ge \mathrm{0,}\\ {A}_{i,t}^{R}+{A}_{i,t}^{R}l{s}_{i,t} & {\rm{if}}\,{s}_{i,t}^{R} < 0.\end{array}$$

Now, as we advanced above, expectations originating from interactions as a recipient govern actions when acting as dictator in the following manner: An individual adapts the donation she is willing to give when playing as a dictator as a consequence of the donation she just received, incorporating an habituation parameter *h* ∈ [0, 1] that, similarly to what occurs with the expectations, balances between their past donation and the donation received (payoff), as shown in eq. 3.

3 $${D}_{i,t+1}^{R}=\mathrm{(1}-h){D}_{i,t}^{R}+h{\pi }_{i,t}.$$

Equations (1)–(3) above define the basic dynamics of our model: higher donations lead to higher aspirations and also to higher donations. With such a model, however, there is no feedback from aspirations to donations and, furthermore, both quantities can take any value in [0, Φ], which is certainly not realistic (practically nobody donates more than Φ/2). Therefore, to prevent these problems, we introduce an additional hypothesis in the model: The donation cannot exceed the amount resulting from subtracting the aspiration of the individual from the endowment; in other words, the amount kept by a dictator after donating is never lower than her aspiration level, which is a sensible assumption. In order to ensure this, we introduce the assumption that donations are bounded:

4 $${D}_{i,t+1}^{R}=\,{\rm{\max }}\,\{0,\,{\rm{\min }}\,[{D}_{i,t+1}^{R},({\rm{\Phi }}-{A}_{i,t+1}^{R})]\}$$

**Figure 1:**
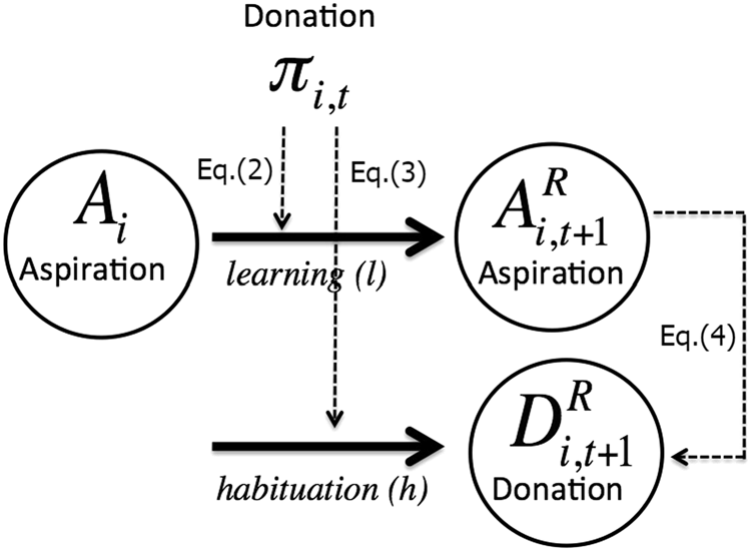
Donations (*π*_*i*,*t*_) affect the aspiration level of recipients (i.e., their expectations about generosity) through a leaning process, Eq. (2). Donations influence also what subjects will donate in future through an habituation process, Eq. (3). In our model, dictators never give more than what they expect to receive as recipients, thus donations never exceed aspiration levels, Eq. (4).

Interestingly, we note that this rule makes learning dependent on *A*_*i*_, thus coupling donations and aspirations as expected. From Eq. (4) it can be seen that high aspirations allow only for small increases in donations, where low aspirations allow more freedom for the evolution of donations according to Eq. (3). This completes the definition of the deterministic version of our model ingredients and their parameterization, and the corresponding dynamics (summarized also in Fig. 1). Without loss of generality, we will choose Φ = 1 for simplicity hereafter. Next, we present the results of our model; the code is available upon request.

## Results

Our analysis of the model is based on extensive simulations with *N* = 1000 individuals. Each simulation run is let to evolve through a transient of 10000 time steps, after which we check whether an stationary state has been reached, defined by the slope of the averaged donation, measured in a time window of 1000 steps, being inferior to 10^−4^. If the system has not reached an stationary state, we let it evolve for subsequent time windows of 1000 steps. Each combination of model parameters has been replicated 100 times and results averaged.

We explore the space of parameters of the learning algorithm, simulating discrete values for *l* ∈ [0, 1] and *h* ∈ [0, 1] at intervals of 0.1, but for the shake of simplicity, we will only present the results at intervals of 0.2. The endowment is set to Φ = 1. Individual aspirations *A*_*i*_ are initialised randomly following a uniform distribution *U*[0, 1] and initial donations are constrained to *D*_*i*_ = Φ − *A*_*i*_, consistently with our model definition. Our aim is to compare the outcomes of our model with the experimental results reported in the literature.

In what follows, we will focus on the general case of the model *l* ∈ [0.2, 0.8] and *h* ∈ [0.2, 0.8], and we will discuss the limiting cases in the Supplementary Information.

### Deterministic model

**Figure 2:**
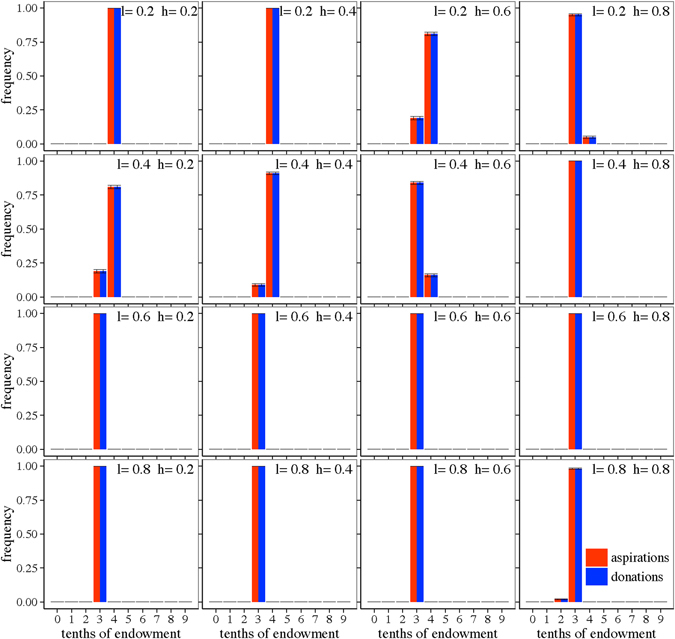
Final averaged distribution of aspirations and donations. Each subplot presents results for a combination of learning rate, *l*, (in increasing order from top to bottom) and habituation parameter, *h*, (in increasing order from left to right). Red bars, histograms of aspirations; blue bars, histograms of donations. Bins labelled *n* of the histograms count the frequency of donations with values verifying (*n*/10) ≤ *D* < (*n* + 1)/10.

Figure 2 shows the distribution of aspirations and donations (rounded to the nearest tenth) at the end of the simulations, averaged over 100 replications, for each combination of parameters *l* and *h*.

The first conclusion we can draw from this figure is that, generically for all values of *l* and *h*, subjects donate nonzero amounts of money and, furthermore, that practically all subjects offer a donation between 30% and 40% of the pie. As for expectations, what we find is that aspiration levels in the population are very similar to the observed donations. Interpreting our results on expectations in terms of social norms, what we observe is a notable level of adherence to such a norm since most subjects are giving a very similar fraction of the pie. We never observer subjects behaving *selfishly* (donating zero), and only in a few cases they exhibit *fair* or *hyper*-*fair* behavior (donating half or more than half of the endowment). We will give a more quantitative characterization of the average parameters of the distributions below.

These results are in general agreement with the experimental observations in so far as most people behave generously, offering nonzero amounts, and also because the mode of the distribution is close to the fair division. Another feature that our model recovers is that aspirations (expectations in real life) are strongly correlated with donations, although this is something that is to be expected as it is built in our premises (agents donate what they expect to receive). However, comparing these findings in more detail to the experimental results^19^, we notice that there is a large discrepancy in terms of the heterogeneity of the distribution of donations. In our simulations described above we find practically all agents at the same level of aspiration and donation, whereas in real life there is a much larger variety of donations and aspirations. It is then clear that, while our deterministic model seems to be capturing the basics of the behavior in DG, we need to introduce some further ingredient in order to reproduce better the empirical results. We address this issue in the following subsection by taking into account the fact that subjects may make mistakes, i.e., designing a stochastic version of our basic model.

### Stochastic model

As we have just stated, the main problem with the results of the deterministic model is the lack of variability. To try to improve our model in this direction, we introduce imperfect decision making (or, as is usually referred to in economics, “trembling hand”), which we implement by adding to the donations a noise term as follows:

5 $${D}_{i,t+1}^{R}=\mathrm{(1}+\varepsilon ){D}_{i,t+1}^{R},$$

where *ε* is drawn from a normal distribution *N*(0,*δ*). In terms of the DG context, this represents the fact that, when making a decision on a donation, people may correct their expectations because they feel that their experience is leading them to overestimate or underestimate the donation arising from the social norm. Alternatively, another reason for such a term is that people may simply feel more or less generous at a given time (realization of the DG) for idiosyncratic reasons. Finally, we could see the noise term as a kind of “rounding” of the values obtained from the update procedure. In any event, we want to stress that this is not the same as learning in so far as depending on the noise the correction of the decision can go against or in favor of the direction marked by the stimulus at each update. An implication of the introduction of noise is that now donations are not slaved to aspirations, and therefore if they still match it would be an additional feature of the experiments that we are reproducing. We will now check whether this new ingredient leads to a distribution of donations less peaked which would be closer to the empirical distributions.

**Figure 3:**
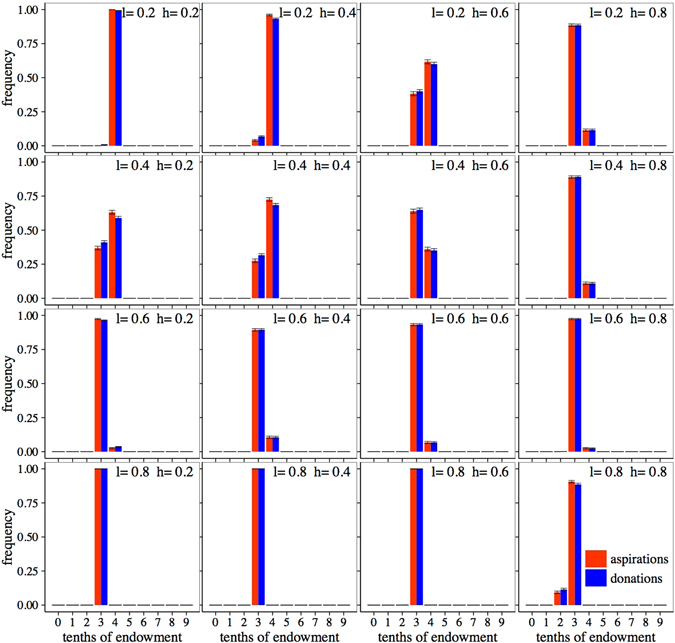
Final averaged distribution of aspirations and donations for *ε* = 0.01. Each subplot presents results for a combination of learning rate, *l*, (in increasing order from top to bottom) and habituation parameter, *h*, (in increasing order from left to right). Red bars, histograms of aspirations; blue bars, histograms of donations. Bins labelled *n* of the histograms count the frequency of donations with values verifying (*n*/10) ≤ *D* < (*n* + 1)/10. Error bars correspond to the standard deviation arising from averaging over 100 realizations.

**Figure 4:**
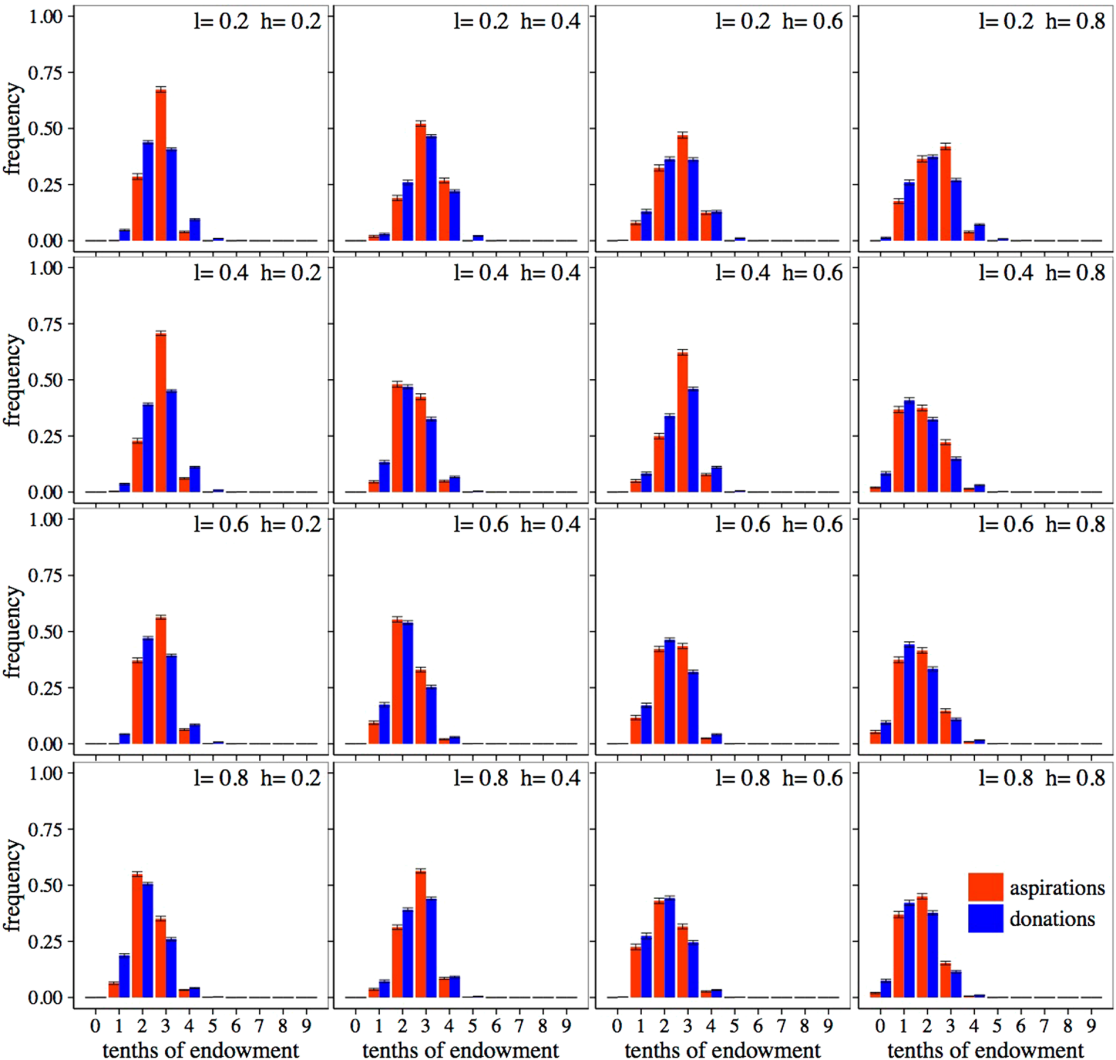
Final averaged distribution of aspirations and donations for *ε* = 0.1. Each subplot presents results for a combination of learning rate, *l*, (in increasing order from top to bottom) and habituation parameter, *h*, (in increasing order from left to right). Red bars, histograms of aspirations; blue bars, histograms of donations. Bins labelled *n* of the histograms count the frequency of donations with values verifying (*n*/10) ≤ *D* < (*n* + 1)/10. Error bars correspond to the standard deviation arising from averaging over 100 realizations.

Figures 3 and 4 present the results for this stochastic version of the model and, as before, we will discuss the general case of the model (*h* ≠ {0, 1} and *l* ≠ {0, 1}). We will first describe our results when the trembling hand effect is very weak, which we represent by choosing *ε* = 0.01. The corresponding final distributions of aspirations and donations, averaged over 100 realizations, are shown in Fig. 3. It can be seen in the majority of the parameter space (0.2 ≤ *h* ≤ 0.8 and 0.2 ≤ *l* ≤ 0.8; limiting cases show different behavior, see Supplementary Information) that donations and aspirations have converged to the same distribution of values in each scenario, with almost all distributions being very sharp, where agents donate and expect to receive between 30% and 40% of the endowment, which is consistent with the average donation found in experiments and also close to the “grand mean” found in the meta study by Engel^12^. However, the results are still very similar to the ones in the general case with no trembling hand (Fig. 2) where the distributions of donations are also peaked and have a mean around the 30% of the endowment. The only minor differences with the deterministic case arise for low values of *l* and low to moderate values of *h*, but even then the donations are basically restricted to two intervals. On the other hand, the very small error bars in our bins indicate that all realizations give approximately the same results, which implies that we are in fact not very far from the deterministic model. As with the deterministic model, we will describe more quantitatively the average parameters of the distributions below.

When we increase the noise component to *ε* = 0.1 and allow the agents to take more imperfect or inaccurate decisions, results are shown in Fig. 4. As advanced above, here we observe distributions widening as noise increases, opposite to the outcome of the limiting cases (see Supplementary Information). It is remarkable that in a large range of the parameter space, simulations yield distributions of donations quite close to the ones found in experimental results (cf. ref. 19). On the other hand, while expectations are still governing the choice of the donated amounts, the correlation is now not exact, also in agreement with the observations from the experiments. In fact, donations are somewhat more spread out than expectations, as was to be expected from the way we introduce the noise in the model. In this case, the results for large habituation, *h* = 0.8, are the ones that reproduce better the experimental results, as they have a small but clearly observable fraction of the population that behaves selfishly, donating nothing. We thus see that our model, when it includes a not so small amount of randomness, reproduces all the main experimental features. In this regard, it is important to point out that other choices for *ε* ∈ [0.05, 0.2] lead to similar histograms, and only when the noise dominates the decisions the model ceases to be a good description of the observations.

### Model extensions

**Figure 5:**
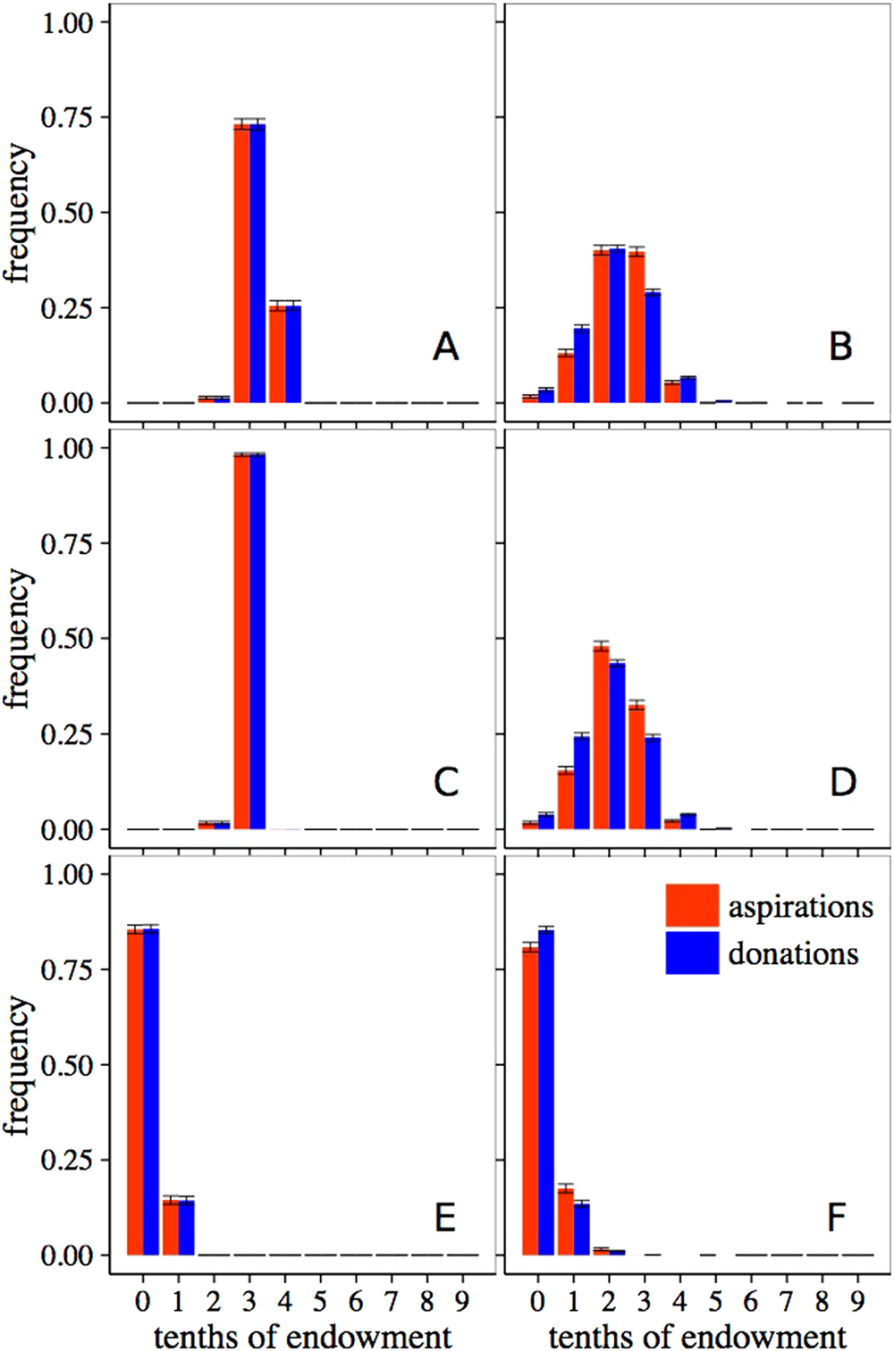
Final averaged distribution of aspirations and donations over all parameters in the general regime. (**A**) Deterministic model, donations 〈*d*〉 = 0.37; G = 0.85. (**B**) Stochastic model with *ε* = 0.1, donations 〈*d*〉 = 0.27; G = 0.71. These two panels are in fact an average of those in Figs 1 and 3. (**C**) Disadvantegous inequality with *ε* = 0, donations 〈*d*〉 = 0.36; G = 0.90. (**D**) Disadvantegous inequality with *ε* = 0.1, donations 〈*d*〉 = 0.25; G = 0.73. (**E**) One free rider, *ε* = 0, donations 〈*d*〉 = 0.06; G = 0.87. (**F**) one free rider, *ε* = 0.1, donations 〈*d*〉 = 0.06; G = 0.86.

In search for more general results, we now consider two additional extensions of the model. First, we look at the effects of having subjects with envious preferences; second, we introduce free-riders in the society, i.e., subjects who always choose to donate zero and never change their strategy. We discuss these two cases separately in the rest of this section. Figure 5 summarizes the comparison of our results above with the two additional variants.

#### Envious individuals and disadvantegeous inequality

This case intends to represent envious or inequality-averse individuals that would never share more than the half of the pie^32^ since they are averse to disadvantegeous distributions. According to this, we impose that donations can never be larger than 50% of the pie (in the limit *D*_*i*_ = *A*_*i*_), this bound reflecting disadvantegeous inequity-aversion.

6 $${D}_{i,t+1}^{R}=\,{\rm{\min }}\,({D}_{i,t+1}^{R}\mathrm{,0.5)}$$

We fix the probability of being inequity-averse, in other words, of behaving as indicated by (eq. 6), to be 0.05, but other percentages of the population lead to very similar behavior. As shown in Fig. 5C,D, the results are very similar to the general case without restrictions: A (deterministic model) and B (stochastic model with *ε* = 0.10). However, comparing panels A with C and B with D, we do observe that the whole distribution is skewed to the left, indicating that the whole society becomes somewhat more selfish. This is not unexpected because there are only a few instances in which donations are larger than half of the pie, and therefore the constraint we have just imposed yields minor modifications of the general behavior.

#### Existence of free-riders

As a second test of the generality of our results, we add free riders —subjects who always donate zero— to our population. Actually, the novel ingredient is not that there are selfish individuals: as we always initialize our simulations randomly, there are some selfish individuals in the previous results. What we are doing now is to generate a separate fraction of the initial population whose donation is zero, and choose the donations of the rest from a uniform distribution as before. The key point here is that the individuals that have been specifically selected to donate zero never update their donation according to their expectations or, in other words, they do not follow any social norm based on them. This amounts to saying that they are not only free-riders, but obstinate free-riders.

Our results in this respect are quite dramatic (see Fig. 5E,F): We find that the introduction of one single free-riding is enough to destroy the social norm since every single player become selfish. This is true for most combinations of parameters, except for low habituation parameter (not shown). Even then, the histogram is largely skewed towards zero donations. The social norm changes from being generous to be completely selfish since all players end up donating zero (and expecting zero). In the case of low values of habituation, subjects are less influenced by the interaction with the free-rider, and are able to maintain positive (but small) expectations and donations. It is also important to note that once a social norm is sufficiently widespread, subjects do not change their behaviour easily, and that is what makes the low habituation distribution less selfish. In any event, as we anticipated, the key mechanism here is that these special free riders refuse to adapt their expectations irrespective of their interactions, and are therefore actively counteracting the establishment of a social norm. This finding supports the view that expectations are fragile, in so far as they need to be constantly confirmed in order to allow subjects to accurately predict the action of others. Observing individuals that constantly go against the norm, like the free-riders we are introducing here, has the effect of making those expectations feeble and unreliable. Interestingly, if subjects that never update their donations have positive values for them, they have a similar capacity to attract the behavior of the rest, showing that what is important is the fact that some people do not follow the norm and not in which direction they go against it. Therefore, learning of all agents arises as a key ingredient to support generosity by avoiding the existence of impenitent free-riders.

## Discussion

In this work we presented a very simple model that explains how people behave in dictator games by introducing the idea that donations are driven from expectations. This mechanism works affecting both our donations, which are modified to be closer to the ones we receive, and our expectations, which also reflect the amounts we actually receive. In turn, expectations affect donations by imposing an upper bound on the amounts we are willing to donate, leading to a coupled evolution of both parameters. With these simple and quite natural assumptions, the model predicts that people will donate sizable amounts of the pie in the stationary regime, while at the same time by construction their expectations will be aligned with their donations. These two results are in excellent agreement with experimental observations^12, 19^. Notwithstanding, we have also found that to recover the diversity of behaviors arising from the experiments we need to introduce some level of noise, or subjects whose hands tremble when they have to decide. When donations are allowed to deviate from expectations between a 5% and a 20%, the corresponding stochastic model predictions are very closely aligned with real behaviors. This in turn makes the connection between expectations and donations less perfect, which is also in good agreement with the observations.

Our model is based on a reinforcement learning dynamics, in which the payoff of an action constitutes a stimulus which agents subsequently use to update their strategies (which, in this specific paper, coincide with their actions). The success of this dynamics in explaining the results of different experiments beyond the current one, namely Prisoner’s Dilemma^33^ or Public Good games^34, 35^ suggests that this type of learning is indeed used by us in many situations. In fact, in ref. 33 it was shown that reinforcement learning was the only rule (among quite a few that have been used in evolutionary models, see ref. 28 for a description of those) that gave rise to a behavior known as moody conditional cooperation (the probability of cooperation is larger when others cooperated and also depends on the subject’s previous choice of cooperation or defection). In addition, reinforcement learning can in fact be a proximate mechanism to explain moody conditional cooperation^35^, as it allows to directly reproduce the experimental results. On the other hand, in terms of the interpretation of reinforcement learning, our two parameters have a clear bearing on actual behavior: The habituation parameter *h* is akin to *herding* (albeit it can also be thought as normative conformity), implying that when *h* is low h agents do not care much about what other people do when they make their own decisions. The learning rate *l* reflects how subjects adapt their expectations to the real environment, with low or high values corresponding to similar adaptability of the agent. While some studies highlight the benefits of these aspects in information acquisition^36, 37^, the fact that our model produces quantitatively correct results as compared with the experiments in ref. 19 for most parameters, but not if one of them is absent or too influential tells us that both processes are also important to reconcile with experimental findings on generosity.

Another interesting point that our research raises touches upon the relation between social norms, behavior and expectations. In the framework introduced by Bicchieri^6^ and discussed in the Introduction, they define social norms as a combination of empirical and normative expectations, in this paper we are confined to the domain of empirical expectations, but our model is certainly suggestive of a social norm actually driving people’s donations in DG experiments. In fact, we believe that the accuracy of our model and its connection to actual social norms can be further tested by additional experiments, in which the normative expectations of the players could also be measured^38^. If normative expectations would coincide with the empirical expectations (as measured in ref. 19 and as explained by our model), we would have shown that we are indeed in the presence of a true social norm. Such an experimental confirmation would clearly establish that a population with a very diverse range of expectations, with agents evolving according to the reinforcement learning paradigm, ends up converging to a commonly shared social norm of generous behavior. This is a promising result and it opens the door to try to look for further models involving social norms in other contexts. At the same time, our result that generous behavior is robust against individuals averse to disadvantageous inequity, but fragile when individuals do not learn (do not update their expectations or do not follow their expectations) means that, first, some degree of social influence may be desirable, and second, violation of social norms must not be tolerated if the good behavior is to be preserved. This conclusion is certainly not new, but it is further evidence supporting the need for some type of punishment or sanctions to avoid free-riding arising from a different evolutionary model such as reinforcement learning. Our results are thus aligned with the work of Bendor and Swistak on the evolution of norms (see ref. 23 and references therein), where they show that social norms arise from evolutionary game theory considerations under boundedly rational behavior. Clearly, the introduction of mechanisms like ostracism^39^ or punishment^40^, possibly incorporating reputation^41^ is a good candidate to solve or at least alleviate this problem, and that would indeed be the case in our model (removed agentes cannot alter the remaining ones’ expectations). Alternatively, direct action on the norm by communication among agents as proposed in ref. 8 can serve to induce generosity while at the same time avoiding sanctions. The fact that most people expect generosity^19^ may then be indeed a consequence of this type of mechanisms having been in action through history.

## Electronic supplementary material

Supplementary Information
