# Supplementary material for: The emergence of altruism as a social norm
Source: Sci Rep. 2017 Aug 29;7:9684. doi: 10.1038/s41598-017-07712-9 (PMC5575094; doi:10.1038/s41598-017-07712-9)
Supplement: Supplementary file 1 — Supplementary Information [file 41598_2017_7712_MOESM1_ESM.pdf]

The emergence of altruism as a social norm

Supplementary Information

María Pereda[1,*], Pablo Brañas-Garza[2], Ismael Rodríguez-Lara[2], and Angel Sánchez[1,3,4]

[1]Grupo Interdisciplinar de Sistemas Complejos, Departamento de Matemáticas, Universidad Carlos III de Madrid, 28911 Leganés, Madrid, Spain
[2]Middlesex University London, Department of Economics, Business School, Hendon Campus, The Burroughs, London NW4 4BT, United Kingdom
[3]Institute UC3M-BS of Financial Big Data, Universidad Carlos III de Madrid, 28903 Getafe, Spain
[4]Institute for Biocomputation and Physics of Complex Systems (BIFI), University of Zaragoza, 50018 Zaragoza, Spain
[*]Corresponding author: mpereda@math.uc3m.es

June 20, 2017

Here focus the discussion of our results by commenting on the limiting cases $h = 0$ and $h = 1$. To begin with, when the habituation h parameter is zero (top panels of figures 1 and 2), the donations of the agents are not directly affected by their aspirations, in so far aspirations only enter by being an upper bound for the donation. This is why the final distribution of donations has its support in values between 0 and 0.5.

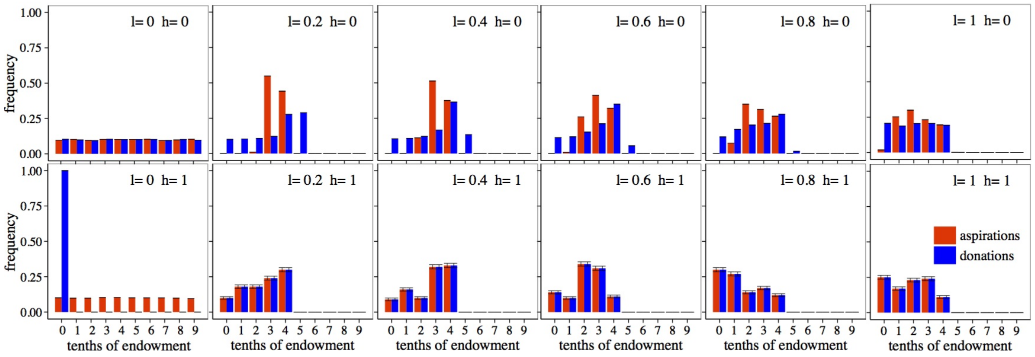

Figure 1: Deterministic model. Final averaged distribution of aspirations and donations for $h = 0$ (top) and $h = 1$ (bottom). Each bin of the histograms counts the frequency of donations with values verifying $(label/10) \leq D < (label + 1)/10$.

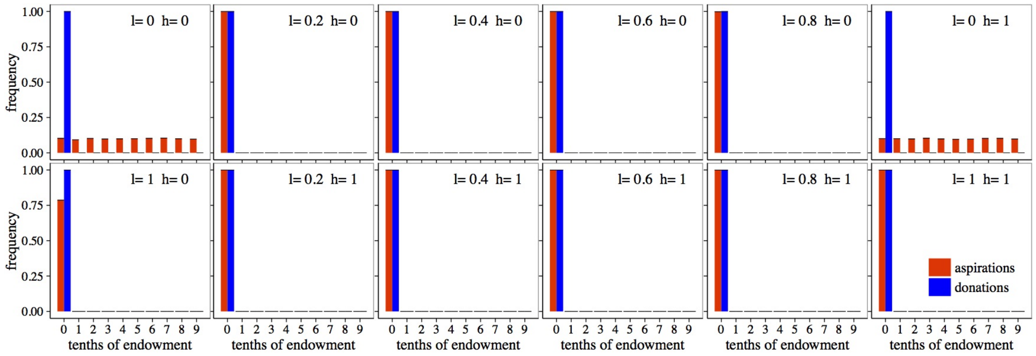

Figure 2: Stochastic model. Final averaged distribution of aspirations and donations for $\varepsilon = 0.1$, $h = 0$ (top) and $h = 1$ (bottom). Each bin of the histograms counts the frequency of donations with values verifying $(label/10) \leq D < (label + 1)/10$.

If $h = 1$, the donations agents make when playing as a dictator in $t+1$ are exactly equal to the payoff they received in the previous step t ($D^R_{i,t+1} = \pi_{i,t}$), so the agents adapt their donations very abruptly, having as a result different distributions of aspirations and donations as a function of the initialisation values. Interestingly, for the deterministic model these two limiting cases give rise to

wide distributions, but for the stochastic model with $\varepsilon = 0.1$, they become peaked around very low values of both donations and aspirations. As we have seen in the main text, exactly the opposite is the case when $h \neq 0, 1$.

Another, different limiting case is $l = 0$ (top panels of figures 3 and 4). This is a peculiar, unrealistic situation in which no learning effect in the aspirations of the agents. As a result, the final aspirations have of course the same values as the initial ones (in our case uniformly distributed). It is important to note that in such a setup, donations go to 0 for all players. While the simulations correspond to an unrealistic case (no learning), the conclusion is that in our model aspirations have to be adaptive if the experimental result that most people behave selflessly is to be recovered.

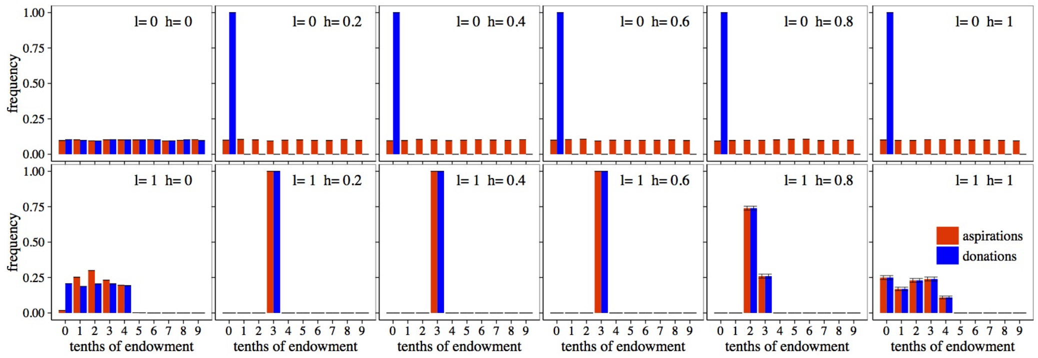

Figure 3: Deterministic model. Final averaged distribution of aspirations and donations for $\varepsilon = 0$ (no trembling hand), $l = 0$ (top) and $l = 1$ (bottom). Each bin of the histograms counts the frequency of donations with values verifying $(label/10) \leq D < (label + 1)/10$.

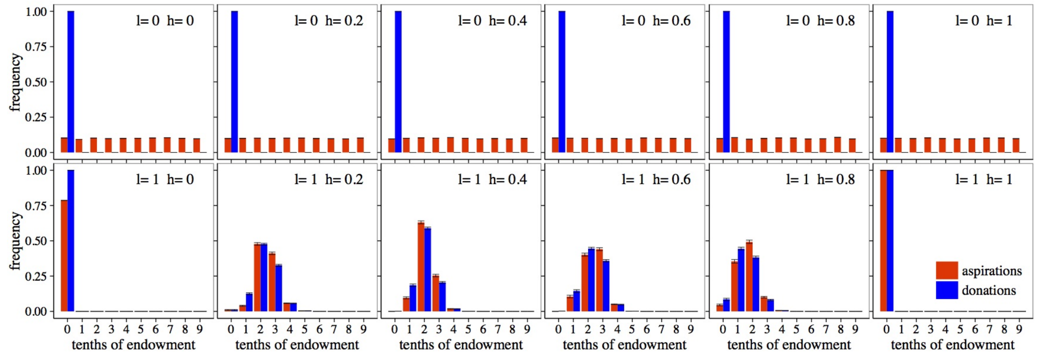

Figure 4: Stochastic model. Final averaged distribution of aspirations and donations for $\varepsilon = 0.1$, $l = 0$ (top) and $l = 1$ (bottom). Each bin of the histograms counts the frequency of donations with values verifying $(label/10) \leq D < (label + 1)/10$.
